# Patient perspectives on key symptoms and preferences for follow-up after upper gastro-intestinal cancer surgery

**DOI:** 10.1007/s00520-022-06922-w

**Published:** 2022-03-11

**Authors:** Philip H. Pucher, Annie Coombes, Orla Evans, Joanna Taylor, Jonathan L. Moore, Annabelle White, Jesper Lagergren, Cara Baker, Mark Kelly, James A. Gossage, Jason Dunn, Sebastian Zeki, Ben E. Byrne, Jervoise Andreyev, Andrew R. Davies, On behalf of the RESTORE study group, Guy’s, St Thomas’ Oesophago-gastric research group

**Affiliations:** 1 Guy’s & St Thomas’ NHS Foundation Trust, London, UK; 2 Portsmouth Hospitals University NHS Trust, Portsmouth, UK; 3 School of Cancer and Pharmaceutical Sciences, King’s College London, London, UK; 4 Department of Molecular Medicine and Surgery, Karolinska Institutet, Stockholm, Sweden; 5 Centre for Surgical Research, University of Bristol, Bristol, UK; 6 United Lincolnshire Hospitals Trust & The School of Medicine, University of Nottingham, Nottingham, UK; 7 Department of Surgery, St Thomas’ Hospital, Westminster Bridge Road, SE1 7EH London, UK

**Keywords:** Survivorship, Symptoms, Esophageal neoplasms, Gastric neoplasms, Esophagectomy, Gastrectomy

## Abstract

**Purpose:**

Long-lasting symptoms and reductions in quality of life are common after oesophago-gastric surgery. Post-operative follow-up has traditionally focussed on tumour recurrence and survival, but there is a growing need to also identify and treat functional sequelae to improve patients’ recovery.

**Methods:**

An electronic survey was circulated via a British national charity for patients undergoing oesophago-gastric surgery and their families. Patients were asked about post-operative symptoms they deemed important to their quality of life, as well as satisfaction and preferences for post-operative follow-up. Differences between satisfied and dissatisfied patients with reference to follow-up were assessed.

**Results:**

Among 362 respondents with a median follow-up of 58 months since surgery (range 3–412), 36 different symptoms were reported as being important to recovery and quality of life after surgery, with a median of 13 symptoms per patient. Most (84%) respondents indicated satisfaction with follow-up. Satisfied patients were more likely to have received longer follow-up (5-year or longer follow-up 60% among satisfied patients vs 27% among unsatisfied, *p* < 0.001). These were also less likely to have seen a dietitian as part of routine follow-up (37% vs 58%, *p* = 0.005).

**Conclusion:**

This patient survey highlights preferences regarding follow-up after oesophago-gastrectomy. Longer follow-up and dietician involvement improved patient satisfaction. Patients reported being concerned by a large number of gastrointestinal and non-gastrointestinal symptoms, highlighting the need for multidisciplinary input and a consensus on how to manage the poly-symptomatic patient.

**Supplementary Information:**

The online version contains supplementary material available at 10.1007/s00520-022-06922-w.

## Introduction

Upper gastro-intestinal cancer resection (oesophagectomy or gastrectomy) represents a major surgical insult that has a major impact on patients’ quality of life [1]. Up to 70% of patients experience long-term symptoms after surgery [2, 3]. Historically, survival rates after surgery for oesophago-gastric cancer were poor, with limited treatment options in the case of disease recurrence. As a result, follow-up practices have focused on survival and the management of tumour recurrence [4, 5]. However, with survival improving, there is a growing need to identify and treat the functional sequelae of oesophago-gastric surgery in order to improve patients’ quality of life [6, 7]. Additionally, with ongoing developments in oncological therapies, the appropriate frequency and duration of follow-up to monitor for disease recurrence remain unknown [8].

There can be little doubt that some patients experience lasting symptoms after oesophago-gastrectomy [9]. Evidence regarding how best to diagnose the underlying cause(s) for these symptoms and how to deliver effective treatment is limited. It is not widely understood that these patients frequently experience multiple symptoms simultaneously which may be caused by a number of co-existing underlying conditions that even experts struggle to predict accurately without diagnostic testing [10]. In the absence of a systematic approach, delays in timely and effective treatment are inevitable and this in turn has a negative effect on the quality of life after surgery. It may also result in a poor allocation of resources with repeated clinic reviews and inefficient diagnostic testing. Quantifying, investigating and treating the symptoms caused by the underlying pathological sequela of oesophago-gastrectomy is distinct from the more general post-operative questionnaires which primarily aim to assess the quality of life (i.e. SF-36).

The RESTORE study (REsolution of SympToms after Oesophago-gastric REsection) aims to establish a consensus on the definition, investigation and management of the symptoms and conditions frequently encountered after upper gastro-intestinal cancer resection. As part of this study, a patient questionnaire was circulated via a patient representative body seeking to assess patients’ experiences, satisfaction and preferences regarding key issues relating to their follow-up after oesophago-gastric surgery. There was a particular focus on symptoms perceived to be important by patients which might inform the subsequent aspects of the study.

## Methods

An electronic survey was circulated via the website of the Oesophageal Patients Association, a British national charity for patients undergoing oesophageal resection and their families. By definition, the survey therefore included patients undergoing oesophago-gastrectomy, almost exclusively for cancer. Patients who had undergone such surgery were invited to respond to the survey (provided in Supplementary Fig. 1). The survey was made available online for a three-week period in March 2021. Survey questions included the year and place of the oesophago-gastric surgery and any additional oncological treatment received. Patients were asked how long they had been, or were due to be, followed up for, what type of specialist performed the majority of follow-up, and whether or not follow-up routinely included review with a dietitian beyond the first post-operative appointment. They were asked how satisfied they felt with their follow-up on a Likert scale of 1–10 and to indicate their preferences for follow-up duration and specialists. Regarding post-operative symptoms, patients were asked to indicate which symptoms they felt were important to overall recovery and quality of life after oesophago-gastrectomy. These were generated from a list of common symptoms as listed in a number of validated questionnaires used in cancer patients and also included free-text fields for patients to add symptoms not already listed [11].

Following local institutional ethics approval, data were retrieved, anonymised and collated.

Results were presented using descriptive statistics. The intention was to compare patients who reported positive experiences with follow-up with the remainder of respondents. Results were dichotomised into satisfied (categorised into Likert scale score 7–10 out of 10) or not satisfied (combining dissatisfied (1–3) and intermediately satisfied (4–6)) groups. These groups were compared for follow-up practices and symptoms using the chi-square or the Mann–Whitney *U* tests. Responses were also grouped and compared to identify patients who wished for follow-up to be shorter, the same, or longer than they had experienced, and those who wished to see a specific specialist as part of their follow-up but did not. There was concern that patients who had undergone surgery within less than 5 years may have indicated follow-up duration responses based on care received so far rather than the clinical care team’s planned duration of follow-up. A secondary analysis was conducted excluding patients within 5 years of surgery; this did not meaningfully change the results or study conclusion and as such full data are presented here. A *p* value of < 0.05 was considered significant. Analysis was performed in IBM SPSS statistics (IBM Corp, IBM SPSS statistics Version 27.0. Armonk, NY: USA).

## Results

### Patients and treatment

A total of 362 responses were received, with a median follow-up time of 58 months since surgery (range 3–412 months). Surgical resection was conducted in 71 different hospitals (which included current UK cancer surgery hospitals, private hospitals and other public hospitals prior to the centralisation of upper gastro-intestinal cancer services in the UK). Perioperative treatments included chemotherapy in 206/362 (57%), radiotherapy in 5/362 (1%) and chemoradiotherapy in 49/362 (14%) of cases. Most patients (302/358, 84%) expressed that they were satisfied with the follow-up (Likert score 7–10).

### Follow-up practices

Patient follow-up was variable but was most commonly performed for 5 years (145/352, 41%) after treatment and by a surgeon (197/358, 55%) (Table 1). Post-operative follow-up of only 1 year or less was reported by 94/352 (26%) of patients. Slightly more than half (198/362, 55%) of patients reported routine dietitian review beyond the first post-operative appointment.

**Table 1:** Patient-reported follow-up practices after oesophago-gastrectomy

	Number	%
Follow-up duration		
> 5 years	48	14%
5 years	145	41%
2 years	67	19%
1 year	46	13%
6 months	28	8%
1 month	18	5%
Primary follow-up speciality		
Surgeon	197	55%
Dietitian / nurse	97	27%
Oncologist	51	14%
Gastroenterologist	13	4%
Routine dietitian review		
Yes	198	55%
No	164	45%
Satisfaction with follow-up		
Positive (Likert 7–10)	302	84%
Neutral (4–6)	37	10%
Negative (1–3)	19	5%

### Patient preferences in follow-up

The majority of patients (275/356, 77%) expressed a preference for follow-up of 5 years or more (Table 2). Comparing follow-up duration received to reported patient preferences, half of the patients (177/362, 49%) felt the follow-up duration they had received was appropriate, whereas 152/362 (42%) wished follow-up was longer. There was no relationship between median time elapsed since surgery and satisfaction. Few patients (33/362, 9%) expressed a desire for shorter follow-up.

**Table 2:** Patient-reported follow-up preferences after oesophago-gastrectomy

	*n*	%
Preferred follow-up duration		
Longer than 5 years	103	29%
5 years	172	48%
2 years	33	9%
12 months	18	5%
6 months	10	3%
1 month	20	6%
Preferred follow-up speciality		
Surgeon	210	57%
Dietitian / nurse	90	23%
Oncologist	51	14%

### Factors affecting satisfaction with follow-up

There was no difference between groups that were unsatisfied and satisfied with the follow-up regarding the time elapsed since treatment or the number of symptoms reported as being important (Table 3). Satisfied patients were more likely to have received longer follow-up (5-year or longer follow-up 60% among satisfied patients vs 27% among unsatisfied, *p* < 0.001). Dissatisfied patients identified follow-up duration as a reason for this (30% of unsatisfied patients were happy with their follow-up duration vs 53% of satisfied patients, *p* = 0.002). There was no difference between groups for which specialists performed the follow-up, but there was a greater proportion of unsatisfied patients who expressed a preference for the follow-up to include a surgeon, whose follow-up was led by a non-surgeon (28% vs 9%, *p* < 0.001). Unsatisfied patients were less likely to have seen a dietitian as part of routine follow-up (37% vs 58%, *p* = 0.005).

**Table 3:** Comparison of patient groups satisfied vs not satisfied with follow-up after oesophago-gastrectomy

	Unsatisfied	Satisfied	*p* value
Months since treatment (months)	50 (4–412)	60 (3–404)	0.498*
Number of reported important symptoms	12 (0–31)	13 (0–33)	0.818*
Duration follow-up received			
1 month 6 months 1 year 2 years 5 years > 5 years	8/55 (15%) 9/55 (16%) 14/55 (25%) 9/55 (16%) 8/55 (15%) 7/55 (13%)	10/293 (3%) 18/293 (6%) 30/293 (9%) 58/293 (20%) 136/293 (46%) 41/293 (14%)	< 0.001**
Desired follow-up duration received	17/56 (30%)	160/302 (53%)	0.002**
Follow-up primarily performed by			
Surgeon Nurse/dietitian Oncologist Gastroenterologist	30/53 14/53 8/53 1/53	165/301 82/301 42/301 12/301	0.892**
Desired but did not see surgeon	16/56 (28%)	27/302 (9%)	< 0.001**
Desired but did not see oncologist	0/56	0/302	1.000**
Desired but did not see nurse or dietitian	4/56 (7%)	32/302 (10%)	0.430**
Dietitian was seen as part of routine follow-up	21/56 (37%)	175/302 (58%)	0.005**

Variation in total sample size due to response fields left empty. Results are reported as median (range) or absolute value with percentages. *Mann–Whitney U test, **Chi-square test

### Symptoms after oesophago-gastrectomy

In all, 36 different symptoms (comprising 31 pre-defined symptoms listed in the questionnaire and 5 additional symptoms added via free-text function by respondents) were reported by patients as being important to recovery and quality of life after oesophago-gastrectomy (Table 4). Some of these included overlapping symptom complexes (e.g. dumping syndrome and dizziness after meals). Symptoms of concern were shared by a large proportion of patients, with all 31 pre-defined symptoms identified by more than 20% of respondents. Patients reported a median of 13 (range 0–33) symptoms they deemed important to their quality of life after surgery. These included both gastrointestinal and non-gastrointestinal symptoms. The most frequent symptoms reported were heartburn (293/362 81%) and early satiety (269/362, 74%).

**Table 4:** Patient-reported important symptoms after oesophago-gastrectomy

Symptom	*n*	%
Heartburn or acid regurgitation	293	80.9
Feeling full after small amount of food	269	74.3
Need to rush to open bowels	252	69.6
Tiredness / lethargy	251	69.3
Difficulty swallowing solids	248	68.5
Weight loss	237	65.5
Abdominal cramps / trapped wind	231	63.8
Reduced appetite	209	57.7
Nausea / feeling sick	209	57.7
Belching or burping	206	56.9
Bowel frequency / consistency	198	54.7
Upper abdominal pain/ discomfort	195	53.9
Dizziness / light headed after meals	193	53.3
Vomiting / being sick/ retching	184	50.8
Lower abdominal pain/ discomfort	181	50.0
Stomach / abdominal gurgling	175	48.3
Difficulty swallowing liquids	158	43.6
Leakage / soiling or lack of control of the bowel	157	43.4
Excessive passing of wind from your bottom	156	43.1
Abdominal bloating / distension	147	40.6
Experienced change in taste	146	40.3
Feeling that you have not emptied your bowel properly	139	38.4
Greasy, pale or oily stool	138	38.1
Woken from sleep to have bowels open	100	27.6
Bleeding from your bottom	93	25.7
Hiccups	84	23.2
Mucus in the stool	84	23.2
Experienced change in smell	79	21.8
Bad breath / halitosis	77	21.3
Itchiness around the bottom	77	21.3
Pain around your bottom	75	20.7
Dumping*	54	14.9
Reflux*	29	8.0
Thoracotomy / rib pain*	17	4.7
Sleep disturbance*	14	3.9
Psychological distress*	11	3.0

^*^Added manually via free-text fields by respondents

## Discussion

This is the first study, to our knowledge, to examine patient preferences and satisfaction with follow-up after oesophago-gastrectomy for cancer. This survey highlights the variability in practice in relation to follow-up duration and the healthcare professionals delivering it. Patients identified a large number of symptoms that they considered important to address as part of the follow-up process. Those reporting lower satisfaction scores received shorter follow-up and were less likely to have seen a surgeon or had regular input from a dietitian.

There is a paucity of evidence in relation to optimal follow-up after oesophago-gastric cancer surgery. One study previously highlighted that follow-up arrangements after cancer treatment in general, which usually involve outpatient appointments at cancer centres, do not meet all cancer survivors’ needs and provide questionable value for money [12]. They highlighted a need to transform cancer care from a ‘one-size fits all’ approach to one based on the assessment of individual needs and preferences. The report of the Independent Cancer Taskforce identified that a large proportion of current cancer costs within the National Health Service (NHS) in the UK relate to treating people who are in the survivorship phase and that more tailored care has the potential to reduce costs through reducing tumour recurrences, better management of side-effects and supporting people to live well [13].

The large number of both gastrointestinal and non-gastrointestinal symptoms reported by patients as ‘important’ after oesophago-gastrectomy is in agreement with the recent LASER study in which 67% of responding patients reported troublesome symptoms at a median of 4.3 years after oesophagectomy [2]. All of the symptoms listed in the present study were felt to be important by 20% or more of participants, thus justifying their inclusion in future studies assessing the symptom burden in this patient group. Given the overlap of symptoms that may be attributed to the varying conditions that commonly affect patients after oesophago-gastrectomy, it remains to be seen whether symptom combinations may be used to predict the underlying cause(s) or whether systematic investigations are required. Either way, this survey forms a patient-led baseline from which a standardised approach to the management of post-operative symptoms may be considered. The proportion of patients reporting concerns over potential mental health symptoms such as sleep disturbance or psychological distress was low and may reflect under-reporting of these issues [3].

Whilst the majority of patients were satisfied with their care overall, this study has demonstrated important differences between this group and the remaining unsatisfied patients. These differences highlight areas that centres might consider when seeking to improve post-treatment follow-up protocols. Patient satisfaction was not associated with time elapsed since treatment, suggesting the risk of recall bias, or satisfaction being related to temporal trends in practice, was low. There were also no differences in the number of symptoms highlighted by satisfied and unsatisfied patients.

The majority of patients expressed a desire to be seen by a surgeon as part of their post-operative care. Understandably, patients feel a strong affiliation to the surgeon who performed their operation, despite the fact that many aspects of symptom management fall outside traditional surgical expertise. Unsurprisingly, dietitian involvement in routine follow-up was higher in patients who reported high satisfaction scores although this did not align with the preferred specialisms involved in follow-up as specified by patients. The reasons for this discordance are unclear. One aspect may relate to the survey design, which did not include descriptions of the roles of various specialties, meaning patients based their responses on their personal experiences alone. Socioeconomic, cultural and educational patient factors have also been shown to play a role in preferences for post-operative follow-up [14]. The importance of dietitian support throughout the surgical pathway is crucial for oesophago-gastrectomy patients, who are at high risk of malnutrition and gastrointestinal complications [15, 16]. Patient understanding of these factors may underlie the preferences reported here to a degree. The fact that only 55% of patients reported routine involvement of a dietitian stands in stark contrast, for example, to a recent Australian and New Zealand survey in which surgeons reported always involving dietetic support postoperatively [16]. Given that the majority of symptoms reported by patients in this study were gastrointestinal in nature, it would seem imperative to increase the rate of dietitian support to manage common symptoms such as reflux or dumping as well as malnutrition. Access to gastroenterology expertise and the means to investigate the underlying conditions that often cause the symptom burden would also be important in the design of any follow-up model.

Patients expressed a preference for longer follow-up. While current practices largely support follow-up for 5 years after surgery [5, 17], there was support (29% of respondents) for the follow-up to continue beyond this, although the preferred duration beyond 5 years was not elucidated in the survey. This reflects the general desire for longer contact with healthcare professionals after complex procedures, as demonstrated in other studies [14, 18, 19]. Other more complex issues such as follow-up ‘as required’, rather than regular pre-set appointments, or future moves towards online symptom reporting triggering follow-up were beyond the remit of this survey, but these issues certainly merit future consideration.

Some methodological limitations of this study deserve discussion. This cross-sectional survey was not fully representative of all oesophago-gastrectomy patients as it excluded those who had not survived or did not take part in the questionnaire. By enrolling patients who self-selected for inclusion via a national patient support group, potential selection bias was introduced. Patient comorbidity or the specifics of treatment (surgical approach or oncological therapy) were not controlled for, with this initial study seeking only to identify a broad scope of patient-identified symptoms. How these relate to specific operations, pathologies and treatments is the aim of future research.

While the patient support association involved in this survey primarily supports patients receiving support for cancer, it is possible that a small number of patients receiving surgery for a benign disease may have been included as they were not explicitly excluded—however, this would be a proportionately small number of respondents. Importantly, patients undergoing minor upper gastro-intestinal surgical procedures were deliberately not captured by the survey which focussed on oesophagectomy. The design of the survey asked patients about their own experiences; responses may have been different if a scenario-based questionnaire was used or if the relative merits of follow-up by different specialists as part of different models were explained as part of the survey. Some questions were asked in an exclusive manner which may not have captured the full scope of the follow-up; the fact that few patients reported that their follow-up care was primarily carried out by a gastroenterologist, for example, must be differentiated from patients where gastroenterologists were additionally consulted for the ongoing management of their symptoms. This may not have been fully captured by the survey.

Follow-up after major cancer surgery has multiple aims including tumour recurrence surveillance, symptom management and patient reassurance. The former was beyond the scope of this study but is clearly important, given recent advances in second and third-line oncological therapies. The overall goal is to restore quality of life after cancer treatment. While this study aimed to characterise the symptoms patients’ deemed important, and their overall satisfaction with follow-up, more work is needed to specifically identify what patients want post-operatively and how this aligns with the medical evidence-base and resources available. Further research is crucial to devising a follow-up regimen that optimises both clinical and patient-reported outcomes after oesophageal surgery.

In conclusion, this large patient survey highlights the important preferences of patients regarding follow-up after surgery for oesophageal or gastric cancer. Routine dietitian involvement was only reported by half of the patients yet was associated with greater patient satisfaction with the follow-up received. Patients were concerned by a large number of gastrointestinal and non-gastrointestinal symptoms, highlighting the need for multidisciplinary input and a consensus on how to best investigate and manage the poly-symptomatic patient.

## Supplementary Information

Below is the link to the electronic supplementary material.

Supplementary file1 (PDF 190 KB)

## Data Availability

All data is available on request.
