# Supplementary material for: Patient perspectives on key symptoms and preferences for follow-up after upper gastro-intestinal cancer surgery
Source: Support Care Cancer. 2022 Mar 11;30(6):5269–75. doi: 10.1007/s00520-022-06922-w (PMC9046299; doi:10.1007/s00520-022-06922-w)
Supplement: Supplementary file 1 — Supplementary file1 (PDF 190 KB) [file 520_2022_6922_MOESM1_ESM.pdf]

Guy's and St Thomas' NHS NHS Foundation Trust AUGIS Association of Upper Gastrointestinal Surgeons of Great Britain and Ireland The Oesophageal Patients Association Making cancer easier to swallow

Patient questions on follow-up after Upper Gastro-intestinal Surgery (part of the RESTORE study)

Thank you for taking the time to answer the questions below. We are trying to understand the common symptoms people experience after major surgery to the oesophagus and stomach and also their perspectives on follow-up after surgery. Your participation is greatly appreciated.

Please circle appropriate answers (all answers will be treated confidentially)

Initials (optional): ……

Date of surgery (Month/year rather than exact date) : …………………………………………….……………

Hospital where surgery took place ? ……………………………………………………………………………………….

Chemotherapy in addition to surgery ? Y / N

Radiotherapy in addition to surgery ? Y / N

How long were you followed-up for at the hospital where you had your surgery ?

1month 6months 12 months 2 years 5years longer

If you were followed-up at another hospital from where you had your surgery, for how long ?

6months 12 months 2 years 5years longer

Who conducted the follow-up mainly ?

Surgeon Oncologist Gastroenterologist Dietitian Nurse (CNS) Other

Did you see a dietitian routinely as part of follow-up beyond the first post-op appointment ? Y / N

Did you have any additional follow-up apart from that with your GP ?

Telephone consultations Email follow-up Other ……………………….……………………………

How happy were you with the Follow-up you received (please circle) ?

1…….2.……3….…4.……5.……6.……7.……8.……9.……10

Very unhappy **Extremely happy**

Ideally, how long would you choose to be followed up after surgery?

1month 6months 12 months 2 years 5years longer

Apart from the treatment you were receiving (e.g seeing Oncology for post-operative chemotherapy) would you have any preference for who conducted your routine follow-up ?

Surgeon Oncologist Nurse / Dietitian Other

We are interested in studying the following symptoms that commonly occur after oesophagectomy or gastrectomy. Please indicate whether you believe the symptom is important (Y/N) and whether any additional symptoms should be included. You do NOT need to have experienced the symptom itself to judge it to be important and are welcome to select all of them if you wish. Please place a "X" in the appropriate box

**Symptom**	**Important Y/N ?** Yes	No	**Symptom**	**Important Y/N ?** Yes	No
Experienced change in smell			Pain around your bottom		
Experienced change in taste			Abdominal bloating/distension		
Bad breath/halitosis			Excessive passing of wind from your bottom		
Difficulty swallowing liquids			Stomach/abdominal gurgling		
Difficulty swallowing solids			Need to rush to open bowels		
Belching or burping			Feeling that you have not emptied your bowel properly		
Heartburn or acid regurgitation			Leakage/ soiling or lack of control of the bowel		
Feeling full after small amount of food			Mucus in the stool		
Reduced appetite			Greasy, pale or oily stool		
Hiccups			Bleeding from your bottom		
Nausea/feeling sick			Itchiness around the bottom		
Vomiting/being sick/retching			Woken from sleep to have bowels open		
Abdominal cramps/trapped wind			Tiredness/lethargy		
Upper abdominal pain/discomfort			Dizzy / light headed after meals		
Lower abdominal pain/discomfort			Bowel frequency / consistency		
Weight loss					
Any other symptoms you feel are important ?					

Thank you for your time. Please send your reply either by email (a scanned document or this word file) to Andrew.davies1@gstt.nhs.uk or by post to Andrew Davies, Consultant Surgeon, Dept of General surgery, East Wing link corridor, St Thomas hospital, London, SE1 7EH

Thank you for your time
